# High circulating miR‐18a, miR‐20a, and miR‐92a expression correlates with poor prognosis in patients with non‐small cell lung cancer

**DOI:** 10.1002/cam4.1238

**Published:** 2017-12-21

**Authors:** Xiaoxiao Xu, Shan Zhu, Zhaowu Tao, Shenglan Ye

**Affiliations:** ^1^ Department of Pneumology The Central Hospital of Wuhan Tongji Medical College Huazhong University of Science and Technology Wuhan 430014 China

**Keywords:** miR‐18a, miR‐20a, miR‐92a, non‐small cell lung cancer (NSCLC)

## Abstract

The purpose of this study was to assess the predictive value of angiogenic miRNAs for disease‐free survival (DFS) and overall survival (OS) of patients with non‐small cell lung cancer (NSCLC). In total, 196 patients with NSCLC (tumor lymph nodes metastasis (TNM) stage I–III) were enrolled and peripheral blood samples were collected. Total RNA was extracted from blood samples, and the relative expression levels of candidate miRNAs were evaluated by real time‐polymerase chain reaction (RT‐PCR). The median follow‐up period was 56.7 months, and the final follow‐up date was in August 2016. The median DFS of all patients was 30.0 (14.0–49.0) months, whereas the median OS was 41.5 (23.0–58.0) months. Furthermore, the 5‐year DFS and OS rates were 11.3% and 32.3%, respectively. Kaplan–Meier (K–M) curves showed that high plasma miR‐18a (*P* < 0.001), miR‐20a (*P* < 0.001), miR‐92a (*P* < 0.001), miR‐126 (*P* < 0.001), miR‐210 (*P* = 0.003), and miR‐19a (*P* = 0.027) expressions levels correlated with a worse DFS. Moreover, patients with high plasma miR‐18a, miR‐20a, miR‐92a, miR‐210, and miR‐126 expression levels had a shorter OS than patients with low expression levels of these miRNAs (all *P* <= 0.001). Furthermore, multivariate Cox regression analyses revealed that high plasma expression levels of miR‐18a, miR‐20a, and miR‐92a as well as lymphatic node metastasis (all *P* < 0.001) were independent risk factors for both DFS and OS in patients with NSCLC. Thus, the circulating miR‐18a, miR‐20a, and miR‐92a levels may serve as novel and promising prognostic biomarkers in patients with NSCLC.

## Introduction

Lung cancer, a major cause of cancer deaths worldwide, has been classified into two subtypes, small‐cell lung cancer (SCLC) and non‐small cell lung cancer (NSCLC). The latter is the most common type of lung cancer, comprising nearly 85% of all lung cancer cases 1, 2. NSCLC is a multifactorial malignancy with several risk factors, such as a history of smoking, exposure to asbestos and malnutrition, which all can increase the incidence of NSCLC 3. Although various treatments are available for patients with NSCLC, such as surgery, chemotherapy and radiotherapy, the 5‐year overall survival (OS) remains low at 15% due to late diagnosis, resistance to cytotoxic agents and a lack of feasible and convincing prognostic biomarkers 4. Therefore, novel and reliable biomarkers to predict the prognosis of NSCLC are urgently needed.

MicroRNAs (miRNAs), which are endogenous non‐coding RNAs, are considered post‐transcriptional regulators of gene expression that contribute to various physiological and pathological process, such as cell proliferation, senescence, apoptosis, and metastasis 5, 6. Many studies have shown that several miRNAs, such as miR‐200, miR‐92c and miR‐18a, play important roles in the tumorigenesis, angiogenesis, and lymphangiogenesis of NSCLC by targeting several angiogenic factors, including VEGF and LATS2 7, 8, 9, 10, 11, 12, 13, 14, 15. However, few clinical studies have investigated the correlation between circulating miRNAs related to angiogenesis and the prognosis of NSCLC. Therefore, the purpose of this study was to assess the predictive value of angiogenic miRNAs for the disease‐free survival (DFS) and overall survival (OS) of patients with NSCLC.

## Methods

### Participants

In total, 196 patients with NSCLC were consecutively enrolled in this study up on admission to the Central Hospital of Wuhan, Tongji Medical College of the Huazhong University of Science and Technology between April 2010 and March 2012. NSCLC was diagnosed based on clinical, radiological and pathological confirmation, and all patients underwent definitive lung surgery for tumors with tumor lymph nodes metastasis (TNM) stages ranging from I to III. Patients who received pre‐operative adjuvant therapies or had a history of other tumors, hematological diseases or severe infection were excluded from this study. This study was approved by the Ethics Committee of the Central Hospital of Wuhan, Tongji Medical College, Huazhong University of Science and Technology and conducted in accordance with the Declaration of Helsinki. All patients signed informed consent forms.

### Sample collection

Peripheral blood was obtained from all patients between 8:00 and 9:00 am the day before surgery. Plasma was subsequently extracted from blood sample and stored at −80°C for miRNA detection.

### MiRNA measurement by real time‐polymerase chain reaction

A panel consisting of 13 pro‐angiogenic miRNAs was selected in this study based on a previous report 16. Total RNA was extracted from plasma samples with a TRIzol LS kit (Takara, Japan). RNA was reverse transcribed using the One Step Primer Script miRNA cDNA Synthesis Kit (Takara, Japan), and the expression levels of these 13 candidate miRNAs were quantitatively analyzed using SYBR Premix Ex TaqTM II (Takara). U6 expression, which served as an internal reference, was used to normalize the expression of miRNAs, and the expression levels of these candidate miRNAs were then calculated with the 2^−∆∆t^ method.

### Baseline data collection and definitions

The baseline characteristics of all patients including age, gender, smoking status, histology, pathological grade, tumor size, lymph node metastasis and TNM stage were collected. The pathological grade was staged as follows: 1‐well differentiated; 2‐moderately differentiated; 3‐poorly differentiated. The TNM stage was assessed based on the sixth edition of the American Joint Committee on Cancer (AJCC) staging manual.

### Follow ups

Patients were followed up at the clinic, at the daytime ward or via telephone call monthly during the first 6 months, once every 3 months until 1 year, and once every 6 months thereafter. The median follow‐up period was 56.7 months, and the final follow‐up date was August 2016. The DFS and OS were calculated.

### Statistics

The data are presented as the mean value ± standard deviation or count (with or without percentage), and groups were compared using the *t* test. Kaplan–Meier (K–M) curves were drawn for the DFS and OS, and the DFS and OS were compared between groups with the log‐rank test. Univariate Cox's proportional hazard regression analyses were performed to evaluate the baseline predictive factors for DFS and OS, and all factors with a *P* value <= 0.1 were further analyzed using a multivariate Cox proportional hazards regression model. All statistical analyses were performed using SPSS software (V21.0, USA). *P* < 0.05 was considered significant.

## Results

### Baseline characteristics

A total of 196 NSCLC patients with a median age of 59.50 (49.19–69.81) years, were enrolled in this study (Table 1). The cohort consisted of 120 (61.2%) males and 76 (38.8%) females, and 86 (43.9%) patients had a history of smoking. Overall 95 (48.5%), 91 (46.4%) and 10 (5.1%) patients harbored adenocarcinoma, squamous cell carcinoma, and other types of tumors, respectively. Moreover, 79 (40.3%) patients had lymph node metastases, whereas the remaining 117 (59.7%) patients did not harbor lymph node metastases. Additional clinicopathological characteristics of these patients with NSCLC are shown in Table 1.

**Table 1:** Baseline characteristics of NSCLC patients

Parameters	NSCLC patients (*N* = 196)
Age (years)	59.50 ± 10.31
Gender (male/female)	120/76
Smoke (*n*/%)	86 (43.9)
Histology	
Adenocarcinoma (*n*/%)	95 (48.5)
Squamous cell carcinoma (*n*/%)	91 (46.4)
Others (*n*/%)	10 (5.1)
Pathological grade	
Poor differentiation (*n*/%)	55 (28.1)
Moderate differentiation (*n*/%)	94 (48.0)
Well differentiation (*n*/%)	47 (24.0)
Tumor size	
>5 cm (*n*/%)	85 (43.4)
<=5 cm (*n*/%)	111 (56.6)
Lymph node metastasis	
Positive (*n*/%)	79 (40.3)
Negative (*n*/%)	117 (59.7)
TNM stage	
III stage (*n*/%)	67 (34.2)
II stage (*n*/%)	65 (33.2)
I stage (*n*/%)	64 (32.7)

Data was presented as mean value ± standard deviation or counts (with or without percentage). NSCLC, non‐small cell lung cancer; TNM stages, tumor lymph nodes metastasis stages.

### DFS and OS in all patients

**Figure 1:**
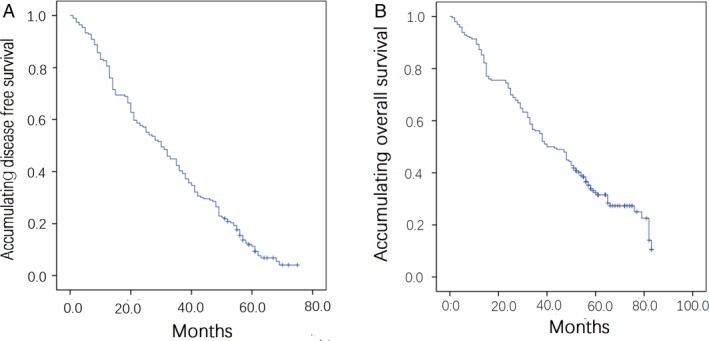
DFS and OS in all patients. (A) DFS in all patients; (B) OS in all patients. Kaplan–Meier (K–M) curves were used to analyse DFS and OS in all patients. DFS, disease‐free survival; OS, overall survival.

As presented in Figure 1, the median DFS of all patients was 30.0 (14.0–49.0) months, whereas median OS was 41.5 (23.0–58.0) months. Moreover, the 5‐year DFS and OS rates were 11.3% and 32.3%, respectively.

### Comparison of candidate miRNAs in patients with NSCLC and health controls

**Figure 2:**
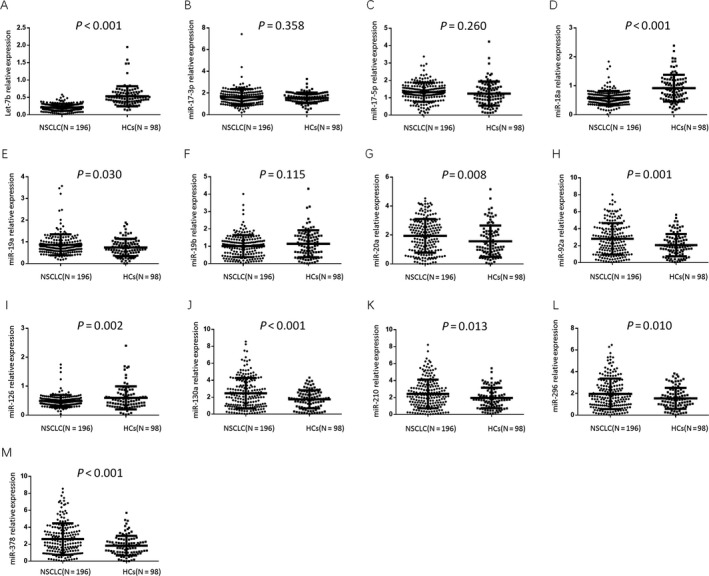
Comparison of candidate miRNAs in patients with NSCLC and HCs. (A) miR‐Let‐7b; (B) miR‐17‐3p; (C) miR‐17‐5p; (D) miR‐18a; (E) miR‐19a; (F) miR‐19b; (G) miR‐20a; (H) miR‐92a; (I) miR‐126; (J) miR‐130a; (K) miR‐210; (L) miR‐296; (M) miR‐378. Groups were compared using the *t* test. *P* < 0.05 was considered significant. NSCLC, non‐small cell lung cancer; HCs, health controls.

The plasma let‐7b (*P* < 0.001, Fig. 2A), miR‐18a (*P* < 0.001, Fig. 2D) and miR‐126 (*P* = 0.002, Fig. 2I) expression levels were lower in patients with NSCLC than in HCs, whereas the expression levels of miR‐19a (*P* = 0.030, Fig. 2E), miR‐20a (*P* = 0.008, Fig. 2G), miR‐92a (*P* = 0.001, Fig. 2H), miR‐130a (*P* < 0.001, Fig. 2J), miR‐210 (*P* = 0.013, Fig. 2K), miR‐296 (*P* = 0.010, Fig. 2L), and miR‐378 (*P* < 0.001, Fig. 2M) were higher in patients with NSCLC than in HCs. The expression levels of the remaining candidate miRNAs did not significantly differ between patients with NSCLC and HCs (Fig. 2).

### Candidate miRNAs between high and low expression groups

The ∆∆Ct and 2^−∆∆Ct^ values of candidate miRNAs were compared between the high expression and low expression group using the *t* test. Both the ∆∆Ct and 2^−∆∆Ct^ between the high expression and low expression groups, and the detailed data are shown in Table 2.

**Table 2:** Candidate miRNAs between high and low expression groups

	2^−∆∆Ct^ Cut‐off value (median)	High expression group				Low expression group				*P* a value	*P* b value
		∆∆Ct mean	∆∆Ct VAR	2^−∆∆Ct^ mean	2^−∆∆Ct^ SD	∆∆Ct mean	∆∆Ct VAR	2^−∆∆Ct^ mean	2^−∆∆Ct^ SD		
let‐7b	0.268	1.199	0.312	0.475	0.249	2.741	0.584	0.167	0.065	<0.001	<0.001
miR‐17‐3p	1.552	−1.006	0.090	2.062	0.602	−0.128	0.263	1.149	0.303	<0.001	<0.001
miR‐17‐5p	1.279	−0.768	0.097	1.747	0.443	0.477	1.005	0.834	0.336	<0.001	<0.001
miR‐18a	0.607	0.152	0.205	0.950	0.350	1.332	0.463	0.428	0.127	<0.001	<0.001
miR‐19a	0.754	−0.097	0.186	1.127	0.453	1.108	0.786	0.517	0.173	<0.001	<0.001
miR‐19b	1.034	−0.594	0.165	1.578	0.543	1.338	1.783	0.524	0.304	<0.001	<0.001
miR‐20a	1.714	−1.432	0.134	2.787	0.732	0.709	2.766	0.846	0.470	<0.001	<0.001
miR‐92a	2.268	−1.923	0.193	3.974	1.255	0.154	1.391	1.147	0.637	<0.001	<0.001
miR‐126	0.471	0.605	0.219	0.700	0.305	1.648	0.508	0.345	0.099	<0.001	<0.001
miR‐130a	1.939	−1.709	0.255	3.487	1.348	0.499	1.873	0.964	0.581	<0.001	<0.001
miR‐210	2.017	−1.724	0.220	3.490	1.228	0.393	2.301	1.047	0.605	<0.001	<0.001
miR‐296	1.557	−1.401	0.260	2.816	1.068	0.637	1.470	0.820	0.439	<0.001	<0.001
miR‐378	2.021	−1.769	0.271	3.649	1.453	0.337	2.369	1.053	0.540	<0.001	<0.001

Comparison among groups was determined by *t* test. *P* < 0.05 was considered significant. VAR, variance; SD, standard deviation.

a Comparison of ∆∆Ct between high expression group and low expression group.

b Comparison of 2^−∆∆Ct^ between high expression group and low expression group.

### Correlation between candidate miRNAs and DFS

**Figure 3:**
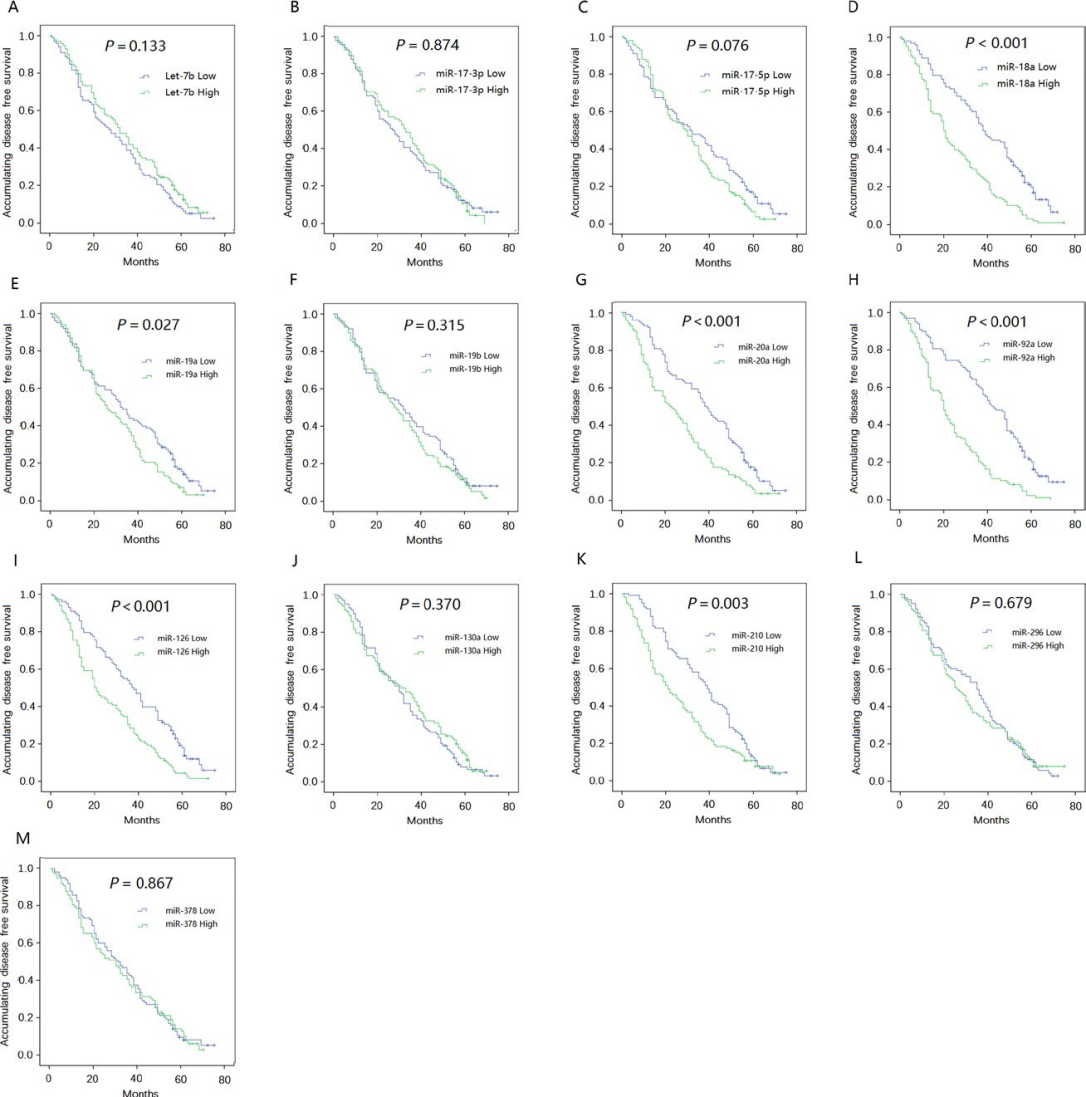
Correlation of candidate miRNAs with DFS. (A) miR‐Let‐7b; (B) miR‐17‐3p; (C) miR‐17‐5p; (D) miR‐18a; (E) miR‐19a; (F) miR‐19b; (G) miR‐20a; (H) miR‐92a; (I) miR‐126; (J) miR‐130a; (K) miR‐210; (L) miR‐296; (M) miR‐378. Kaplan–Meier curves and log‐rank test were used to evaluate the correlation between candidate miRNAs and DFS. *P* < 0.05 was considered significant. DFS, disease‐free survival.

Our study classified candidate miRNAs into high expression and low expression groups according to the median 2^−∆∆Ct^ value. As listed in Figure 3, low plasma expression levels of miR‐18a, miR‐20a, miR‐92a, and miR‐126 correlated with prolonged DFS (all *P* < 0.001). Similarly, low plasma expression levels of miR‐210 (*P* = 0.003) and miR‐19a (*P* = 0.027) were associated with longer DFS compared with high expression. The remaining candidate miRNAs did not correlate with DFS.

### Correlation of candidate miRNAs with OS

**Figure 4:**
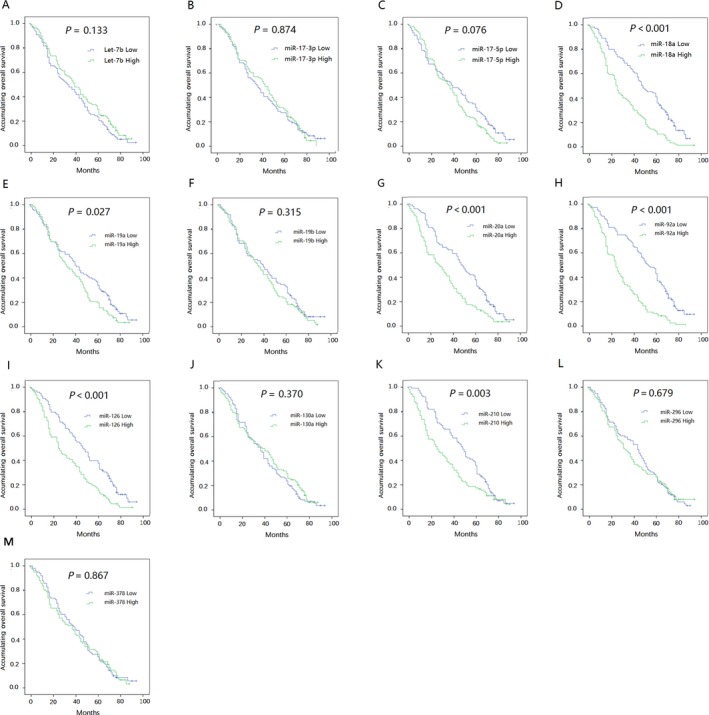
Correlation of candidate miRNAs with OS. (A) miR‐Let‐7b; (B) miR‐17‐3p; (C) miR‐17‐5p; (D) miR‐18a; (E) miR‐19a; (F) miR‐19b; (G) miR‐20a; (H) miR‐92a; (I) miR‐126; (J) miR‐130a; (K) miR‐210; (L) miR‐296; (M) miR‐378. Kaplan–Meier curves and log‐rank test were used to evaluate the correlation between candidate miRNAs and OS. *P* <* *0.05 was considered significant. OS, overall survival.

Patients with high plasma miR‐18a (*P* < 0.001), miR‐20a (*P* < 0.001), miR‐92a (*P* < 0.001), miR‐210 and miR‐126 (*P* = 0.001) expression levels exhibited a shorter OS compared with patients expressing low levels of these miRNAs (Fig. 4), whereas the remaining candidate miRNAs were not associated with OS.

### Predictive factors analysis for DFS

Univariate Cox proportional hazards regression analyses were used to evaluate the baseline predictive factors for DFS, as presented in Table 3, which indicated that high plasma expression levels of miR‐18a (*P* < 0.001), miR‐19a (*P* = 0.031), miR‐20a (*P* < 0.001), miR‐92a (*P* < 0.001), miR‐126 (*P* < 0.001) and miR‐210 (*P* = 0.003) were associated with shorter DFS. Furthermore, a higher pathological grade (*P* = 0.007), tumor size >5 cm (*P* = 0.005), lymph node metastasis (*P* < 0.001), and higher TNM stage (*P* = 0.001) also predicted shorter DFS. All factors with a *P* value <0.1 were further analyzed with a multivariate Cox proportional hazards regression model, which revealed that high plasma expression levels of miR‐18a (*P* < 0.001), miR‐20a (*P* < 0.001), miR‐92a (*P* < 0.001), and miR‐126 (*P* = 0.004) as well as lymphatic node metastasis (*P* < 0.001) were independent risk factors for poor DFS.

**Table 3:** Predictive factors analysis for DFS in NSCLC patients

Parameters	Univariate Cox				Multivariate Cox			
	*P* value	HR	95% CI		*P* value	HR	95% CI	
			Lower	Higher			Lower	Higher
Let‐7b	0.141	0.802	0.599	1.076	–	–	–	–
miR‐17‐3p	0.877	0.977	0.728	1.310	–	–	–	–
miR‐17‐5p	0.083	1.300	0.967	1.747	0.460	1.122	0.826	1.525
miR‐18a	**<0.001**	2.143	1.587	2.893	**<0.001**	1.917	1.390	2.644
miR‐19a	**0.031**	1.386	1.031	1.863	0.223	1.217	0.887	1.670
miR‐19b	0.324	1.159	0.864	1.554	–	–	–	–
miR‐20a	**<0.001**	1.792	1.333	2.409	**<0.001**	1.885	1.379	2.577
miR‐92a	**<0.001**	2.528	1.863	3.429	**<0.001**	2.028	1.466	2.806
miR‐126	**<0.001**	1.867	1.386	2.515	**0.004**	1.582	1.158	2.161
miR‐130a	0.379	0.876	0.653	1.176	–	–	–	–
miR‐210	**0.003**	1.555	1.159	2.086	0.072	1.341	0.975	1.844
miR‐296	0.685	1.063	0.793	1.425	–	–	–	–
miR‐378	0.869	1.025	0.765	1.374	–	–	–	–
Age (years)	0.051	1.016	1.000	1.032	0.368	1.007	0.991	1.024
Gender (male)	0.239	1.199	0.887	1.621	–	–	–	–
Smoking	0.848	1.029	0.766	1.383	–	–	–	–
Higher pathological grade^a^	**0.007**	1.344	1.085	1.665	0.083	1.228	0.974	1.548
Tumor size >5 cm	**0.005**	1.541	1.143	2.078	0.679	0.916	0.604	1.389
Lymph node metastasis	**<0.001**	2.638	1.943	3.580	**<0.001**	2.684	1.738	4.145
Higher TNM stage^b^	**0.001**	1.379	1.147	1.658	0.643	0.936	0.706	1.240

Data was presented as *P* value, Hazard Ratio (HR) and 95% CI. Univariate Cox proportional hazard regression was used to analyze baseline factors affecting DFS, while factors with *P* value <0.1 was further determined by multivariate Cox proportional hazard regression. Bold *P* value <0.05 was considered significant.

a Pathological grade was scored as 1‐well differentiation, 2‐moderate differentiation, 3‐poor differentiation.

b TNM stage was divided into 1–4 scores in accordance with Stage I–IV (No stage IV patients enrolled in this study). NSCLC, non‐small cell lung cancer; DFS, disease‐free survival; TNM stages, tumor lymph nodes metastasis stages.

### Predictive factors analysis for OS

High plasma expression levels of miR‐18a (*P* < 0.001), miR‐20a (*P* < 0.001), miR‐92a (*P* < 0.001), miR‐126 (*P* = 0.002), and miR‐210 (*P* < 0.001) were associated with a shorter OS (Table 4). Furthermore, a high pathological grade (*P* = 0.017), tumor size >5 cm (*P* < 0.001), lymph node metastasis (*P* < 0.001), and a high TNM stage (*P* < 0.001) also correlated with poor OS in patients with NSCLC. Based on the multivariate Cox proportional hazards regression model, high plasma expression levels of miR‐18a (*P* = 0.000), miR‐20a (*P* = 0.000), miR‐92a (*P* = 0.000), and miR‐210 (*P* = 0.004) as well as lymph node metastasis (*P* = 0.000) were independent risk factors for shorter OS in patients with NSCLC.

**Table 4:** Predictive factors analysis for OS in NSCLC patients

Parameters	Univariate Cox				Multivariate Cox			
	*P* value	HR	95% CI		*P* value	HR	95% CI	
			Lower	Higher			Lower	Higher
Let‐7b	0.412	0.870	0.625	1.213	–	–	–	–
miR‐17‐3p	0.522	0.897	0.643	1.251	–	–	–	–
miR‐17‐5p	0.099	1.326	0.949	1.852	0.259	1.226	0.860	1.747
miR‐18a	**<0.001**	2.257	1.601	3.181	**0.000**	2.116	1.484	3.018
miR‐19a	0.141	1.284	0.920	1.792	–	–	–	–
miR‐19b	0.188	1.251	0.897	1.744	–	–	–	–
miR‐20a	**<0.001**	2.052	1.463	2.880	**0.000**	2.303	1.596	3.325
miR‐92a	**<0.001**	2.844	2.003	4.038	**0.000**	2.127	1.469	3.080
miR‐126	**0.002**	1.706	1.218	2.388	0.118	1.320	0.932	1.871
miR‐130a	0.518	0.896	0.643	1.250	–	–	–	–
miR‐210	**<0.001**	2.135	1.520	2.998	**0.004**	1.706	1.183	2.462
miR‐296	0.198	1.244	0.892	1.735	–	–	–	–
miR‐378	0.605	1.092	0.783	1.522	–	–	–	–
Age (years)	0.096	1.014	0.997	1.032	0.555	1.006	0.987	1.024
Gender (male)	0.204	1.253	0.885	1.773	–	–	–	–
Smoking	0.168	1.268	0.905	1.776	–	–	–	–
Higher pathologicagrade^a^	**0.017**	1.344	1.055	1.713	0.261	1.162	0.894	1.511
Tumor size >5 cm	**<0.001**	2.032	1.452	2.844	0.213	1.320	0.853	2.043
Lymph node metastasis	**<0.001**	3.289	2.337	4.628	**0.000**	2.476	1.536	3.992
Higher TNM stage^a^	**<0.001**	1.559	1.267	1.920	0.724	0.944	0.684	1.302

Data was presented as *P* value, Hazard Ratio (HR) and 95% CI. Univariate Cox proportional hazard regression was used to analyze baseline factors affecting OS, while factors with *P* value <0.1 was further determined by multivariate Cox proportional hazard regression. Bold *P* value <0.05 was considered significant.

Pathological grade was scored as 1‐well differentiation, 2‐moderate differentiation, 3‐poor differentiation.

a TNM stage was divided into 1–4 scores in accordance with Stage I–IV (No stage IV patients enrolled in this study). NSCLC, non‐small cell lung cancer; OS, overall survival; TNM stages, tumor lymph nodes metastasis stages.

## Discussion

In this study, we found that high plasma expression levels of miR‐18a, miR‐19a, miR‐20a, miR‐92a, miR‐126, and miR‐210 correlated with a shorter DFS and/or OS. Furthermore, only the plasma miR‐18a, miR‐20a, and miR‐92a levels as well as lymph node metastasis were independent risk factors for both DFS and OS in patients with NSCLC.

In terms of histological types, NSCLC is classified into adenocarcinoma, squamous‐cell carcinoma and large‐cell carcinoma 1. Recent evidence have proven that tumor growth and metastasis could lead to a poor prognosis in patients with NSCLC by pathological angiogenesis that forms new abnormal and poorly organized vessels based on the pre‐existing vascular network 17, 18, 19. The VEGF family includes major pro‐angiogenic factors, such as VEGF‐(A‐D) and placenta growth factor (PIGF), which bind to transmembrane receptor tyrosine kinases (RTKs) to control signal transduction that results in endothelial proliferation, metastasis and the generation of new vessels 20, 21. Accumulating evidence has shown that exosomes serve as local and systemic cell‐to‐cell mediators of oncogenic information by horizontally transferring several bioactive molecules to contribute to carcinoma progression, including mRNAs and protein 22. Exosomes of tumor cells contain many biologically stable miRNAs, that may serve as convincing diagnostic and prognostic biomarkers 23. Upon cell apoptosis or cell rupture, these miRNAs may be released from tumor cells into blood, allowing aberrant miRNA expression to be examined in blood samples, which may reflect tumor progression 24. Therefore, we extracted plasma candidate miRNAs from peripheral blood in this study.

MiRNAs, binding to 3′‐untranslated regions (3′‐UTRs), repress gene and protein expression to regulate cellular function 25. Moreover, dysregulated miRNA expression mediates oncogenes or tumor repressors to affect tumorigenesis and carcinoma prognosis 26. Accumulating evidence demonstrated that miRNAs affect tumor angiogenesis and prognosis in patients with NSCLC by downregulating pro‐angiogenic factors by repressing various signaling pathways, such as the VEGF or c‐Met/phosphoinosmde‐3‐kinase (PI3k)/Akt/mammalian target of rapamycin (mTOR) pathway 10, 27, 28. A pervious study revealed that the miR‐17‐92 cluster may be a convincing biomarker for the early detection of gastric cancer 29. In our study, we selected 13 pro‐angiogenic miRNAs by analyzing a previous report 16. We used K‐M curves and log‐rank tests to evaluate the correlation of these candidate miRNAs with DFS and OS, which suggested that miR‐18a, miR‐20a and miR‐92a, miR‐126, miR‐210, and miR‐19a overexpression was associated with shorter DFS, whereas miR‐18a, miR‐20a, miR‐92a, miR‐126, and miR‐210 were correlated with worse OS. Subsequently, we used univariate and multivariate Cox regression analyses to assess predictive factors analysis for DFS and OS, which revealed that only miR‐18a, miR‐20a, and miR‐92a independently predicted both DFS and OS in patients with NSCLC.

We enrolled 98 age‐adjusted normal subjects in another time period, and then we obtained their blood samples and examined relevant indicators which were compared with the expression levels of the 13 candidate miRNAs in patients with NSCLC. This comparison demonstrated that the let‐7b, miR‐18a, and miR‐126 expression levels were lower in patients with NSCLC, whereas the miR‐19a, miR‐20a, miR‐92a, miR‐130a, miR‐210, miR‐296, and miR‐378 expression levels were higher in patients with NSCLC than in HCs.

MiR‐18a, a crucial member of the miR‐17‐92 family, promotes the tumorigenesis and tumor angiogenesis 30. Accumulating evidence has demonstrated that miR‐18a plays an oncogenic role in colon cancer, breast cancer and prostate cancer 30, 31, 32, and miR‐18a upregulation induces cell proliferation by stimulating cyclin D1 via the PTEN‐PI3K‐AKT‐mTOR signaling axis in oesophageal squamous cell carcinoma cells 33. Moreover, miR‐18a expression also accelerates cell invasion, promotes G1/S phase cell cycle arrest and enhances the action of pro‐apoptotic agents in colorectal cancer cells 34. In clinical studies, miR‐18a expression strongly correlated with clinical TNM stage, tumor differentiation and regional lymph node metastasis (*P* < 0.005), and miR‐18a upregulation is negatively associated with the clinical response of NSCLC because it decreases the sensitivity of cells to radiation by activating the serine/threonine‐protein kinase 4 (STK4) pathway 15. Furthermore, high miR‐18a expression was correlated with a high recurrence rate in hepatocellular carcinoma (HCC) by promoting pathological angiogenesis via an increase in VEGFA expression 35. In accordance with previous findings, our study found that high plasma miR‐18a expression correlated with shorter survival, and was an independent risk factor for DFS and OS in patients with NSCLC. This finding may be due to two possible reasons. The overexpression of miR‐18a promotes tumor growth and metastasis by regulating angiogenic factors to increase cell proliferation and cell invasion. Alternatively, miR‐18a may reduce the sensitivity of cells to radiation treatment 15, 33, 34, 35.

MiR‐20a, which is also part of the miR‐17‐92 cluster, has been shown to promote various cancers, such as lung cancer, gastric cancer and hepatocellular carcinoma, by enhancing cell proliferation and promoting cell migration 36, 37, 38. Moreover, miR‐20a is also considered to act as a tumor promoter in colorectal cancer by increasing the levels of the pro‐apoptotic factor BID, which is associated with the tumor necrosis factor‐related apoptosis‐inducing ligand (TRAIL) sensitivity 39. In nasopharyngeal cancer, the overexpression of miR‐20a stimulated radio‐resistance by targeting Rab27B to regulate cell apoptosis and radiosensitivity 40. However, few clinical studies have evaluated the correlation between circulating miR‐20a and the prognosis of patients with NSCLC. We found that high plasma miR‐20a expression, was associated with shorter DFS and OS in patients with NSCLC and consequently served as an independent risk factor. This finding may be because high miR‐20a expression is associated with tumor size and migration because it participates in cell proliferation, cell apoptosis and invasion by targeting multiple pathways, such as BID and TRAIL. In addition, the overexpression of miR‐20a promoted radio‐resistance in patients with NSCLC, which may contribute to poor prognosis, as demonstrated in other cancers 39, 40.

MiR‐92a has been described as a cancer‐related miRNA. Specifically, the overexpression of miR‐92a plays an oncogenic role in osteosarcoma cells in nude mice and stimulates tumor growth by targeting the phosphatase and tensin homolog/AKT (PTEN/AKT) signaling pathway 41. MiR‐92a also induces cell proliferation and invasion by targeting F‐box and WD repeat domain‐containing 7 (FBXW7) in cervical cancer 42. Moreover, the overexpression of miR‐92a was associated with worse survival in a mouse model of intraperitoneal ovarian cancer, because it targeted signal transducer and activator of transcription 3 (STAT3) signaling 43. Lastly, high miR‐92a expression correlates with worse OS in patients with gastric cancer, suggesting that miR‐92a is a reliable prognostic biomarker in patients with gastric cancer 44. However, little was known about the relationship between the plasma miR‐92a levels and survival in patients with NSCLC. The results of this study show that high plasma miR‐92a expression was associated with worse DFS and OS and independently predicted prognosis in patients with in NSCLC. The mechanism underlying this relationship may be an association between miR‐92a and a more severe disease condition or drug resistance as well as a similarity to miR‐18a and miR‐20a 41, 42, 43.

Additionally, this study also found that plasma miR‐126 and miR‐210 upregulation correlated with shorter DFS or OS in patients with NSCLC. Various studies have shown that these two miRNAs affect cell survival, metastasis, and differentiation 16, 45. Specifically, miR‐210 and miR‐126 overexpression induces angiogenesis by stimulating VEGF production or targeting HIF 44, 46.

However, our study was also subjected to the following limitations. (1) These candidate miRNAs were only assessed in plasma samples and not compared with expression in tumour tissue samples; (2) Patients with stage IV NSCLC were not enrolled in our study; (3) The sample size was relatively small; (4) The detailed mechanisms by which these miRNAs promote angiogenesis in NSCLC were not investigated in the current study; (5) Whole blood miRNA experiments and studies of exosomal miRNA in the same patient are necessary, and these experiments are planned in a future study.

In conclusion, circulating miR‐18a, miR‐20a, and miR‐92a may act as novel and promising prognostic biomarkers in patients with NSCLC.

## Conflicts of Interest

None declared.
